# Nutrition label literacy and adolescent mental well-being: a three-wave longitudinal study of healthy eating self-efficacy and physical activity as a moderator

**DOI:** 10.3389/fnut.2026.1906815

**Published:** 2026-08-14

**Authors:** Jiale Wang, Xianqun Zuo, Huiteng Xu, Li Luo, Yekang Ma

**Affiliations:** 1 School of Physical Education, Xinjiang Normal University, Urumqi, China; 2 Department of Physical Education, Woosuk University, Wanju-gun, North Jeolla Province, Republic of Korea; 3 Faculty of Management, Shinawatra University, Bang Toei, Pathum Thani, Thailand

**Keywords:** adolescents, healthy eating self-efficacy, mental well-being, nutrition label literacy, physical activity

## Abstract

**Background:**

Nutrition label literacy may help adolescents interpret packaged-food information and develop confidence in healthier food choices, but its longitudinal association with positive mental well-being remains insufficiently understood. This study examined whether healthy eating self-efficacy statistically mediated the prospective association between nutrition label literacy and mental well-being among Chinese adolescents and whether physical activity moderated the second stage of this pathway.

**Methods:**

A three-wave longitudinal survey was conducted among Chinese adolescents aged 12–18 years. Data were collected at T1 in November 2025, T2 in January 2026, and T3 in March 2026. The final matched sample included 2,412 adolescents. Nutrition label literacy was assessed at T1, healthy eating self-efficacy at T1 and T2, mental well-being at T1 and T3, and physical activity at T2. Longitudinal mediation and moderated mediation were examined using PROCESS Model 14 logic with 5,000 bootstrap resamples, adjusting for age, gender, only-child status, residence, T1 healthy eating self-efficacy, and T1 mental well-being.

**Results:**

T1 nutrition label literacy was positively associated with T2 healthy eating self-efficacy (*β* = 0.231, *p* < 0.001). T2 healthy eating self-efficacy was positively associated with T3 mental well-being (*β* = 0.201, *p* < 0.001) in the longitudinal mediation model. The indirect association from T1 nutrition label literacy to T3 mental well-being through T2 healthy eating self-efficacy was significant (*β* = 0.046, 95% CI [0.036, 0.058]). In the moderated mediation model, the T2 healthy eating self-efficacy × T2 physical activity interaction was significant (*β* = 0.122, *p* < 0.001), and the index of moderated mediation was significant (index = 0.028, 95% CI [0.020, 0.037]). Conditional indirect effects were stronger at higher levels of physical activity.

**Conclusion:**

Nutrition label literacy may be prospectively associated with adolescent mental well-being partly through healthy eating self-efficacy, and higher physical activity was associated with a stronger indirect pathway. Because the study was observational, these findings represent longitudinal associations rather than causal effects. These findings suggest that integrated school-based strategies combining nutrition label education, healthy eating self-efficacy enhancement, and physical activity promotion are promising, but their effectiveness should be evaluated in future intervention studies.

## 1 Introduction

Adolescence is a developmental period in which health-related capacities, dietary routines, autonomy in food choice, and psychological functioning become increasingly consolidated (1). The broader Lancet Commission has similarly framed adolescent health as a foundation for lifelong development (2). Nutrition is especially important during adolescence because growth, school demands, peer influence, body-image concerns, and expanding independence intersect during this period (3). Young people now make food choices in environments marked by packaged foods, persuasive marketing, convenience-oriented eating, and competing nutrition messages (4). Global child nutrition reports have also emphasized that contemporary food environments expose young people to unhealthy commercial food systems (5). At the same time, physical activity is recognized as a core lifestyle behavior that supports adolescent physical and mental health (6). Food processing and the expansion of ultra-processed products make food-label interpretation more important for adolescents who must judge nutritional quality under real-world constraints (7). School and family contexts can further shape whether nutrition information is understood, trusted, and translated into everyday choices (8).

Nutritional health literacy is broader than factual nutrition knowledge because it concerns access to, understanding of, appraisal of, and application of food-related information in concrete decision-making situations (9). Food literacy adds a practical dimension by emphasizing how individuals plan, select, prepare, and consume food within social and environmental constraints (10). Scoping work has emphasized that food literacy is multidimensional and varies across planning, selection, preparation, and consumption domains (11). A systematic review also notes that nutrition literacy and food literacy are related but not identical constructs (12). Chinese school-age children now have a validated food and nutrition literacy framework, indicating that food-related literacy can be assessed meaningfully in this population (13). Evidence from Malaysian adolescents further shows that food label literacy is a relevant adolescent nutrition construct and that label reading does not guarantee high-level label comprehension (14). European label research further shows that consumer responses depend not only on information availability but also on comprehension and context (15). The present study therefore focused on nutrition label literacy as a specific, task-based capacity within the broader field of nutritional health literacy. In this study, nutrition literacy refers to the capacity to obtain, understand, and use nutrition-related information in decision-making situations; food literacy emphasizes the practical planning, selection, preparation, and consumption of food; and nutrition label literacy denotes the narrower, task-based ability to read, compare, and interpret the information presented on packaged-food labels (9, 10, 12). The term nutrition label literacy is therefore used throughout this manuscript, rather than the broader term health literacy, because the construct measured here reflects task-based label comparison and interpretation rather than the full breadth of accessing, appraising, and applying health information in personal and social contexts.

### 1.1 Mental well-being

Mental well-being was selected as the dependent variable because the study focused on positive adolescent health rather than only psychological symptoms. From a social cognitive perspective, health-promoting beliefs and actions are relevant to well-being because they shape perceived control, adaptive motivation, and everyday self-regulation (16). In the present study, mental well-being was measured with the WHO-5, a short index of positive feelings, vitality, and interest in daily life (17). The WHO-5 has also been validated in adolescent and clinical youth samples, supporting its use as a short positive well-being measure (18). Cross-cultural adolescent validation work further supports the use of the WHO-5 in youth research contexts (19). The Chinese version of the WHO-5 has demonstrated acceptable reliability and validity in mainland Chinese samples (20). Psychological well-being research more broadly indicates that positive affect, vitality, and daily engagement are important components of healthy functioning (21). Health psychology also treats well-being as a meaningful outcome that may be influenced by lifestyle, perceived control, and social conditions (22).

### 1.2 Nutrition label literacy and mental well-being

Nutrition label literacy may be linked to mental well-being through several indirect and developmentally plausible routes. Adolescents who can interpret label information may experience greater confidence in food choice, greater perceived control over eating, and stronger orientation toward health-promoting routines. Health literacy theory emphasizes that people need not only health information but also the capacity to use that information in personal and social contexts (23). Integrated models of health literacy further highlight the movement from access and understanding toward appraisal, communication, and action (24). Updated evidence on health literacy also links limited literacy with poorer health outcomes across populations (25).

In youth research, health literacy is embedded in school, family, community, and media environments rather than located only in individual knowledge (26). Reviews of adolescent health literacy have linked literacy to health behaviors, service use, and psychosocial outcomes, but the effects are typically modest and shaped by context (27). Adolescent health literacy frameworks further stress that literacy develops through interaction between young people and their health-information environments (28). Lifespan health-literacy scholarship has called for context-sensitive approaches to youth health literacy (29). On this basis, nutrition label literacy may be prospectively associated with mental well-being, but this association should be understood as indirect and non-deterministic rather than as a direct causal effect.

### 1.3 Healthy eating self-efficacy as the mediator

Healthy eating self-efficacy was specified as the mediator because nutrition label understanding does not automatically translate into healthier choices or broader well-being. Adolescent eating behavior is influenced by personal preferences, peer norms, family meals, school food availability, convenience, and marketing exposure (30). Qualitative evidence has shown that adolescents often describe taste, convenience, time, price, and social situations as more immediate influences than abstract nutrition knowledge (31). Social cognitive theory provides a broader account of how cognitive capabilities, self-beliefs, and social context jointly shape behavior (32). Nutrition education theory similarly emphasizes that knowledge needs to be linked with skills, motivation, and behavioral opportunities (33). These contextual pressures make self-efficacy theoretically important because it reflects whether adolescents believe they can apply nutrition information under everyday constraints.

Healthy eating self-efficacy may therefore serve as the psychological bridge between label-related competence and later well-being. A prospective study found that diet quality was associated with later mental health among adolescents, supporting a temporally ordered diet–mental health link (34). Systematic evidence suggests that diet and mental health are related in children and adolescents, although effect sizes and causal interpretations remain cautious (35). Reviews focused on diet and depression in youth have reported associations but emphasized heterogeneity (36). Canadian longitudinal evidence has recently linked sugar-sweetened beverage and fruit-and-vegetable intake with subsequent adolescent mental health and well-being outcomes (37). Peer-support research suggests that eating-related self-efficacy is connected with adolescents’ food-intake patterns in everyday social settings (38). School-based healthy eating interventions also show that educational strategies are more promising when they address multiple determinants rather than knowledge alone (39).

### 1.4 Physical activity as the moderator

Physical activity was examined as a moderator because it may provide a broader lifestyle context in which healthy eating self-efficacy is more likely to be associated with later well-being. Adolescents with higher physical activity may experience greater vitality, mastery, emotion regulation, stress recovery, and perceived bodily competence, all of which can support the translation of health-related confidence into more favorable psychological functioning (40). Reviews of physical activity and adolescent mental health suggest that active lifestyles are generally associated with more favorable mental health outcomes, although evidence quality varies (41). This position is consistent with youth physical activity research showing that movement behaviors are related not only to fitness and weight-related indicators but also to cognitive, emotional, and psychosocial outcomes (42). Therefore, physical activity was not treated as a competing mediator in the present model but as a behavioral resource that may condition whether confidence in healthy eating is accompanied by subsequent well-being.

The second-stage moderation hypothesis is also consistent with a multidomain lifestyle perspective. Healthy eating confidence and physical activity often develop in shared school, family, and peer contexts, and both behaviors require self-regulation in the face of time pressure, convenience-oriented food environments, academic demands, and social influence. Objective-measure reviews show that physical activity is associated with multiple health indicators in school-aged youth (43). Evidence on school-aged youth also links physical activity and fitness with broad health benefits (44). When adolescents are physically active, healthy eating self-efficacy may operate within a broader pattern of self-regulated health behavior rather than as an isolated belief. In this context, confidence in choosing healthier foods may be more likely to coexist with positive affect, energy, and daily engagement. By contrast, when physical activity is low, healthy eating self-efficacy may remain psychologically beneficial but may have a weaker connection with later well-being because other lifestyle resources are limited.

This study therefore specified physical activity as a second-stage moderator of the T2 healthy eating self-efficacy to T3 mental well-being pathway. The model does not assume that physical activity changes the earlier association between T1 nutrition label literacy and T2 healthy eating self-efficacy. Instead, it tests whether the positive longitudinal association between healthy eating self-efficacy and later mental well-being differs across levels of T2 physical activity. This placement is theoretically aligned with the idea that nutrition education and physical activity promotion may work together as complementary components of adolescent health promotion rather than as separate intervention domains (45). Given that moderation effects in longitudinal observational studies are usually small, the expected interaction was interpreted cautiously as a conditional association rather than as evidence of a causal protective effect (46). Several considerations supported this second-stage moderator specification rather than treating physical activity as a mediator, a confounder, or only an independent predictor. First, physical activity was measured at T2, after T1 nutrition label literacy, so it could not confound the association between the T1 predictor and the T2 mediator, and its main effect on T3 mental well-being was still estimated in the model. Second, there is no strong theoretical reason to expect that label-comparison skill itself would produce substantial changes in adolescents’ overall exercise volume within a few months, which argues against specifying physical activity as an additional mediator in this particular model. Third, social cognitive and multidomain lifestyle perspectives suggest that physical activity functions as a broader behavioral context that can condition whether health-related confidence is accompanied by later well-being, which corresponds statistically to moderation of the second-stage path rather than to an additional indirect path.

### 1.5 Current study

**Figure 1:**
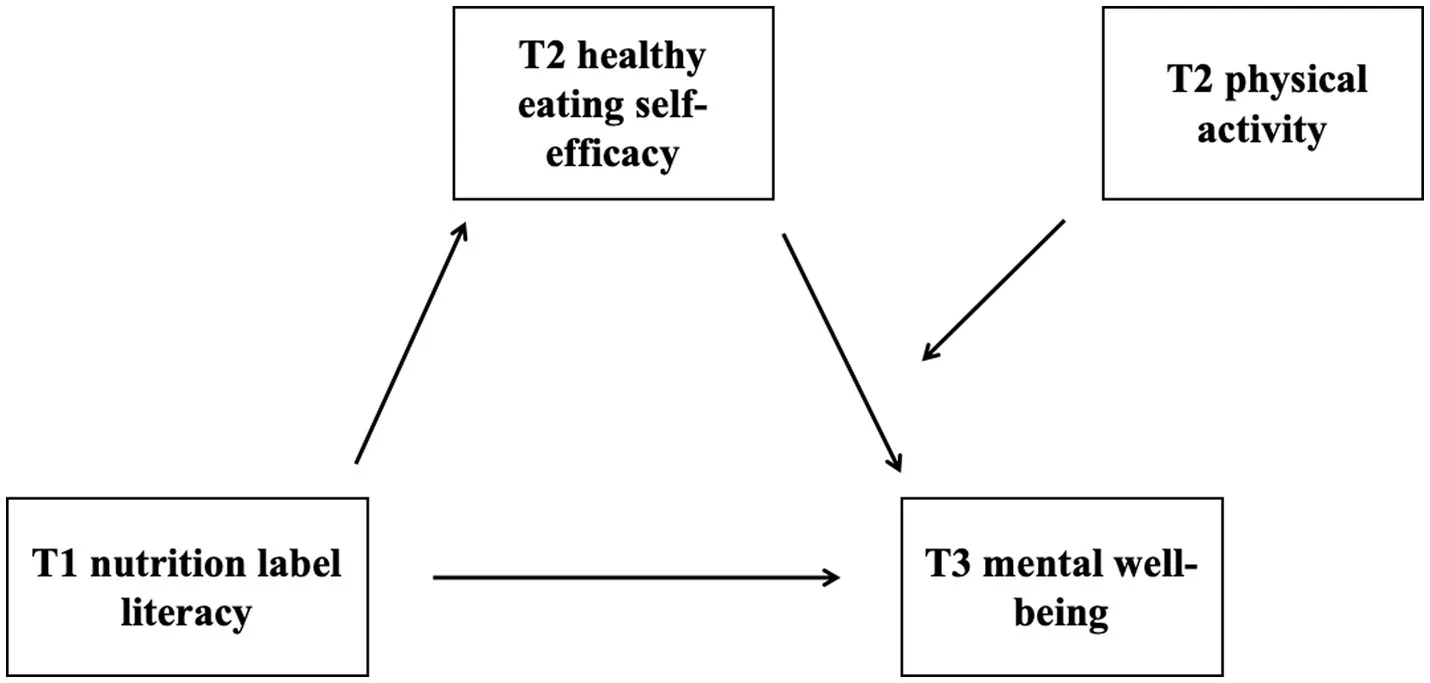
Hypothesized longitudinal moderated mediation model.

A review of the existing literature did not identify a fully identical four-variable model, although closely related studies have examined nutrition literacy, label understanding, self-efficacy, diet quality, physical activity, and adolescent well-being separately. Cross-sectional evidence is available, but longitudinal moderated mediation studies in child and adolescent samples remain relatively scarce. Specifically, previous studies have established that nutrition literacy and food label understanding are related to dietary choices, that eating-related self-efficacy is associated with adolescent food-intake patterns, and that diet and physical activity are each associated with youth mental health. However, it remains unknown whether task-based nutrition label literacy is prospectively associated with later mental well-being, whether healthy eating self-efficacy operates as an intermediate psychosocial pathway over time, and whether this indirect pathway varies across levels of physical activity. A longitudinal moderated mediation design is needed to address these questions because it separates the predictor, the mediator, and the outcome in time, controls for baseline levels of the mediator and the outcome, and tests whether the indirect association is conditional rather than uniform. The present study therefore tested a three-wave longitudinal moderated mediation model among Chinese adolescents. The model was grounded in health literacy theory, social cognitive theory, health-promotion thinking, and multidomain lifestyle health-promotion frameworks. T1 nutrition label literacy was specified as the predictor, T2 healthy eating self-efficacy as the mediator, T3 mental well-being as the outcome, and T2 physical activity as the moderator of the T2 self-efficacy to T3 well-being path. Age, gender, only-child status, residence, baseline healthy eating self-efficacy, and baseline mental well-being were included as covariates. The hypothesized model is shown in Figure 1.

H1: T1 nutrition label literacy will be positively associated with T3 mental well-being after controlling for demographic variables, T1 healthy eating self-efficacy, and T1 mental well-being.

H2: T1 nutrition label literacy will be positively associated with T2 healthy eating self-efficacy.

H3: T2 healthy eating self-efficacy will mediate the longitudinal association between T1 nutrition label literacy and T3 mental well-being.

*H4*: T2 physical activity will moderate the association between T2 healthy eating self-efficacy and T3 mental well-being, such that the association will be stronger at higher levels of physical activity.

*H5*: The indirect association between T1 nutrition label literacy and T3 mental well-being through T2 healthy eating self-efficacy will vary across levels of T2 physical activity.

## 2 Materials and methods

### 2.1 Participants and procedure

This study used a three-wave longitudinal survey design among Chinese adolescents aged 12–18 years. The survey was conducted through an online questionnaire at three time points: T1 in November 2025, T2 in January 2026, and T3 in March 2026. Participants were recruited from school classes using a convenience sampling approach. Specifically, adolescents were recruited from 10 schools located in central, northern, and southern China. These schools were invited because they had an established research collaboration with the authors’ team, and whole classes within each participating school were invited to take part. Because the sample was a convenience sample drawn from collaborating schools rather than a probability sample, it is not nationally representative, and the possibility of selection bias is addressed explicitly in the Limitations section. The first page of the questionnaire presented the study information, voluntariness of participation, anonymity, confidentiality, and the right to withdraw. Participants who did not agree to participate were not allowed to proceed to the questionnaire. No names, identity card numbers, home addresses, or other direct identifiers were collected. An anonymous tracking code was used to match responses across the three waves.

At T1, 3,214 questionnaires were collected; at T2, 2,714 questionnaires were collected; and at T3, 2,412 questionnaires were collected. Matching was conducted using the anonymous tracking code. The final matched sample included 2,412 adolescents, corresponding to a final retention rate of 75.05% from T1 to T3. The final sample included 1,157 boys (47.97%) and 1,255 girls (52.03%), with a mean age of 14.32 years (SD = 1.75).

The study was approved by the Biomedicine Ethics Committee of Xinjiang Normal University before the initiation of the project. All procedures were conducted in accordance with the Declaration of Helsinki. Because all participants were adolescents, written informed consent was obtained from both the adolescent participants and their legal guardians before participation.

### 2.2 Measures

#### 2.2.1 Nutrition label literacy

Nutrition label literacy was measured at T1 with a 10-item task-based nutrition label comparison test. The item format was adapted from the Food Label Literacy for Applied Nutrition Knowledge questionnaire, which uses food-label comparison tasks to assess whether children can identify healthier packaged-food options (47). Because the original tool was developed in a United States food-label context, the present study contextualized the tasks for Chinese packaged-food labels and age-appropriate adolescent reading levels. The Chinese adaptation also drew on the food and nutrition literacy framework developed for Chinese school-age children to ensure that the test reflected nutrition knowledge, label interpretation, and food-choice skills relevant to the local food environment. Each item was scored as 1 for a correct response and 0 for an incorrect or uncertain response, yielding a total score from 0 to 10. Higher scores indicated stronger nutrition label literacy. Because the instrument was a task-based knowledge test rather than a reflective psychological scale, reliability was evaluated using KR-20 and item difficulty/discrimination indices. In the present study, KR-20 was 0.747. Item analysis in the final matched sample further indicated that item difficulty (the proportion of correct responses) ranged from 0.46 to 0.80 and that corrected item-total (point-biserial) discrimination indices ranged from 0.40 to 0.43, suggesting acceptable difficulty levels and adequate discrimination for a brief task-based test; the item-level indices for all 10 items are reported in Table A1. As preliminary construct-validity evidence, test scores were positively and significantly correlated with healthy eating self-efficacy assessed at T2 (*r* = 0.244), physical activity assessed at T2 (*r* = 0.146), and mental well-being assessed at T3 (*r* = 0.185) (see Table 1).

**Table 1:** Correlations among covariates and core study variables.

Variable	Age	Gender	Only_child	Urban	Grade	T1_NLL	T1_HESE	T2_HESE	T1_WHO5	T3_WHO5	T2_PA
Age	—										
Gender	−0.021	—									
Only_child	0.018	−0.028	—								
Urban	−0.021	−0.044*	0.011	—							
Grade	0.917***	−0.010	0.021	−0.022	—						
T1_NLL	0.009	0.006	0.011	0.127***	0.005	—					
T1_HESE	0.016	−0.121***	0.021	0.016	0.005	0.136***	—				
T2_HESE	0.023	−0.053**	0.032	0.016	0.037	0.244***	0.503***	—			
T1_WHO5	−0.041*	−0.078***	0.058**	0.088***	−0.057**	0.094**	0.200***	0.058**	—		
T3_WHO5	−0.007	−0.057**	0.048*	0.036	−0.011	0.185***	0.204***	0.255***	0.538***	—	
T2_PA	0.021	−0.015	0.020	0.038	0.025	0.146***	0.125***	0.243***	0.094***	0.166***	—

Lower triangular Pearson correlations are shown. *p < 0.05; **p < 0.01; ***p < 0.001. Gender: 1 = male, 2 = female; only_child: 1 = yes; urban: 1 = urban.

#### 2.2.2 Healthy eating self-efficacy

Healthy eating self-efficacy was assessed at T1 and T2 with a brief four-item measure of adolescents’ confidence in making healthier food choices in everyday situations. The items were adapted from adolescent healthy eating and healthy food choice self-efficacy measures used in previous youth nutrition research (48). These brief self-efficacy items are consistent with social cognitive approaches to adolescent dietary behavior because they assess perceived capability rather than actual dietary intake. Given the three-wave survey design and the need to reduce respondent burden, a short form was used. Items were rated on a 5-point scale from 1 = strongly disagree to 5 = strongly agree, and item scores were averaged. Higher scores indicated greater healthy eating self-efficacy. No reverse-coded items were used. The same four items and the same response format were administered at T1 and T2, and longitudinal measurement invariance across the two waves was formally tested (see Section 3.3). Cronbach’s alpha was 0.762 at T1 and 0.743 at T2.

#### 2.2.3 Mental well-being

Mental well-being was assessed at T1 and T3 with the five-item WHO-5 Well-Being Index. The WHO-5 is a brief measure of positive well-being that assesses good spirits, relaxation, vitality, feeling rested, and interest in daily life during the past 2 weeks. The WHO scoring framework uses a 6-point response format from 0 = at no time to 5 = all of the time, producing a raw score from 0 to 25, with higher scores indicating better mental well-being (49). The WHO-5 has demonstrated acceptable psychometric performance in Chinese samples and youth validation studies. In particular, the Chinese version of the WHO-5 has shown acceptable reliability and construct validity in mainland Chinese samples (20), and adolescent validation studies support its use as a brief positive well-being measure in youth populations (18, 19), supporting the cultural applicability of the instrument to Chinese adolescents. Additional school-aged child validation evidence supports the suitability of WHO-5 indices for younger populations (50). In the present study, Cronbach’s *α* values were 0.769 at T1 and 0.793 at T3.

#### 2.2.4 Physical activity

Physical activity was assessed at T2 using the Physical Activity Rating Scale-3 (PARS-3), a brief instrument commonly used in Chinese student research (51). The scale contains three items assessing exercise intensity, duration, and frequency. Following the conventional Chinese scoring approach, total physical activity volume was calculated as intensity × (duration − 1) × frequency, with possible scores ranging from 0 to 100. Scores of 19 or below indicate low physical activity, scores from 20 to 42 indicate moderate physical activity, and scores of 43 or above indicate high physical activity. PARS-3 was used as a continuous moderator in the main moderated mediation model and as a categorical descriptive index in threshold analyses. Because PARS-3 is a multiplicative behavioral index combining intensity, duration, and frequency rather than a reflective psychometric scale, internal consistency coefficients were not calculated.

#### 2.2.5 Covariates

Age, gender, only-child status, and residence were included as demographic covariates. Gender was coded as 1 = male and 2 = female. Only-child status was coded as 1 = yes and 0 = no. Residence was coded as 1 = urban and 0 = rural. T1 healthy eating self-efficacy and T1 mental well-being were also included as baseline covariates in the longitudinal models.

### 2.3 Data analysis

Data were analyzed using Python 3.13, statsmodels 0.14.5, IBM SPSS Statistics, and IBM SPSS AMOS. Descriptive statistics were computed for all core variables, and reliability was evaluated using coefficients appropriate to each measure. KR-20 was used for the task-based nutrition label literacy test, Cronbach’s alpha was used for healthy eating self-efficacy and WHO-5, and internal consistency was not calculated for PARS-3 because it is a formative behavioral index. Attrition analyses compared adolescents who completed all three waves with those who did not using t tests or chi-square tests, with Cohen’s d and Cramer’s V used as effect-size indicators. Missing values, out-of-range values, duplicate tracking codes, attention-check failures, and implausibly short response times were examined before the main analyses.

Common method bias was examined using Harman’s single-factor test, which is widely used as an initial diagnostic procedure in self-report behavioral research (52). Measurement validity was assessed using confirmatory factor analysis for T1 healthy eating self-efficacy, T2 healthy eating self-efficacy, T1 mental well-being, and T3 mental well-being. Model fit was evaluated using CFI, TLI, RMSEA, and SRMR according to conventional structural equation modeling criteria (53). CFA model fit was also interpreted with reference to commonly used cutoff criteria in covariance structure analysis (54). Composite reliability and average variance extracted were calculated to evaluate convergent validity (55). Discriminant validity was examined using the heterotrait-monotrait ratio (56). In addition, longitudinal measurement invariance of healthy eating self-efficacy across T1 and T2 was examined by sequentially comparing configural, metric, and scalar models within a two-factor longitudinal confirmatory factor analysis that allowed correlated uniquenesses for identical items across waves; changes in model fit were evaluated using the Chi-square difference test together with commonly used change-in-fit criteria (ΔCFI ≤ 0.010 and ΔRMSEA ≤ 0.015).

Longitudinal mediation and moderated mediation were tested using the logic of PROCESS Model 14, which is appropriate when the second-stage path from the mediator to the outcome is moderated (57). This regression-based framework, rather than a full latent-variable structural equation model or a cross-lagged panel model, was retained for the primary analysis for three reasons. First, the index of moderated mediation and the conditional indirect effects that constituted the focal estimands of this study are directly defined and bootstrapped within this framework (57, 58). Second, the predictor was a task-based test score and the moderator was a formative behavioral index combining exercise intensity, duration, and frequency, neither of which is appropriately specified as a reflective latent variable, so a full latent-variable model would have required mixing reflective and formative measurement components. Third, the measurement quality of the reflective scales was evaluated separately through confirmatory factor analysis, composite reliability, and longitudinal measurement invariance testing, and the substantive pattern of results was robust across sensitivity analyses. We nevertheless acknowledge that observed-score regression does not fully correct for measurement error, and this is noted as a limitation. The predictor was T1 nutrition label literacy, the mediator was T2 healthy eating self-efficacy, the outcome was T3 mental well-being, and the moderator was T2 physical activity. Age, gender, only-child status, residence, T1 healthy eating self-efficacy, and T1 mental well-being were included as covariates. Indirect effects were estimated using 5,000 bootstrap samples and 95% confidence intervals (59). The index of moderated mediation was used to evaluate whether the indirect association varied across levels of physical activity (58). The longitudinal design was interpreted in line with methodological guidance that warns against causal overinterpretation of mediation in observational data (60). Bias in cross-sectional mediation analyses has been noted in methodological work, supporting the use of temporally ordered mediation when available (61). Longitudinal mediation guidance further emphasizes clear measurement timing and baseline control (62). Indirect effects were interpreted using established mediation analysis principles (63). Missing data and attrition were considered in line with applied missing-data guidance (64). Bootstrapping and robust interpretation were used because nonnormal variables can affect conventional standard errors (65). Continuous variables involved in interaction terms were mean-centered before products were created. Variance inflation factors were examined to assess multicollinearity. All statistical tests used a two-sided significance level of *p* < 0.05.

## 3 Results

### 3.1 Attrition analysis and participant characteristics

The initial T1 sample included 3,214 adolescents. A total of 2,714 adolescents participated at T2, and 2,412 participated at T3. The final matched sample included 2,412 adolescents, yielding a final retention rate of 75.05% from T1 to T3. The final sample ranged from 12 to 18 years of age (M = 14.32, SD = 1.75) and included 1,157 boys and 1,255 girls. Participant characteristics are shown in Table 2.

**Table 2:** Participant characteristics in the final matched sample.

Characteristic	*n*/value	%/description
T1 sample	3,214	100.00%
T2 sample	2,714	84.44% of T1
T3 sample/final matched sample	2,412	75.05% of T1
Age, years, M (SD)	14.32 (1.75)	range 12–18
Gender: male	1,157	47.97%
Gender: female	1,255	52.03%
Only child: yes	819	33.96%
Only child: no	1,593	66.04%
Residence: urban	725	30.06%
Residence: rural	1,687	69.94%

Percentages are based on the final matched sample unless otherwise indicated.

Attrition analyses indicated that completers and non-completers did not differ significantly in age, T1 nutrition label literacy, T1 mental well-being, gender, or residence. Completers reported slightly higher T1 healthy eating self-efficacy than non-completers, and only-child status differed slightly between completers and non-completers. However, the corresponding effect sizes were small (Table 3).

**Table 3:** Attrition analysis comparing completers and non-completers.

Variable	Completers	Non-completers	Test statistic	p	Effect size
Age	14.32 (1.75)	14.28 (1.74)	0.60	0.547	d = 0.025
T1 NLL	5.81 (2.67)	5.83 (2.72)	−0.21	0.831	d = −0.009
T1 HESE	2.91 (0.83)	2.76 (0.84)	4.41	<0.001	d = 0.182
T1 WHO-5	13.42 (4.17)	13.18 (4.10)	1.42	0.155	d = 0.058
Gender	Categorical	Categorical	χ^2^ = 0.05	0.824	V = 0.004
Only-child status	Categorical	Categorical	χ^2^ = 3.96	0.047	V = 0.035
Residence	Categorical	Categorical	χ^2^ = 0.01	0.933	V = 0.001

Completers were adolescents with valid matched data across all three waves. Continuous variables are shown as M (SD).

### 3.2 Data quality, descriptive statistics, reliability, and normality

No duplicate tracking codes were identified within any wave, and no item-level missing values or out-of-range values were observed for the matched core variables. The PARS-3 total score was recalculated from intensity, duration, and frequency, and all recorded PARS-3 total scores matched the specified formula. In the full wave-specific datasets, 199 T1 responses, 192 T2 responses, and 180 T3 responses were completed in less than 120 s. In the final matched sample, 92 adolescents failed the attention-check item at T1. These cases were retained in the main analysis and examined in sensitivity analyses.

Descriptive statistics, reliability, skewness, and kurtosis are presented in Table 4. The nutrition label literacy test showed acceptable reliability for a brief task-based test. Healthy eating self-efficacy and mental well-being also showed acceptable internal consistency. PARS-3 was right-skewed, which is common for self-reported physical activity; therefore, bootstrap estimation and category-based sensitivity checks were used. In addition, threshold distributions for lower mental well-being, physical activity categories, and nutrition label literacy levels are shown in Table 5.

**Table 4:** Descriptive statistics, reliability, and normality indices.

Variable	Range	M	SD	Skewness	Kurtosis	Reliability
T1 nutrition label literacy	0–10	5.81	2.67	−0.285	−0.858	KR-20 = 0.747
T1 healthy eating self-efficacy	1–5	2.91	0.83	0.047	−0.524	α = 0.762
T2 healthy eating self-efficacy	1–5	3.04	0.79	0.033	−0.370	α = 0.743
T1 mental well-being	0–25	13.42	4.17	−0.048	−0.208	α = 0.769
T3 mental well-being	0–25	14.44	4.38	−0.066	−0.311	α = 0.793
T2 physical activity	0–100	28.72	24.87	1.069	0.301	Not applicable

NLL, nutrition label literacy; HESE, healthy eating self-efficacy; WHO-5, mental well-being; PA, physical activity. PARS-3 is a behavioral index and internal consistency was not calculated.

**Table 5:** Threshold distributions for key variables.

Indicator	n	%
WHO-5 < 13 at T1	994	41.21%
WHO-5 < 13 at T3	808	33.50%
Low physical activity (≤19)	1,144	47.43%
Moderate physical activity (20–42)	720	29.85%
High physical activity (≥43)	548	22.72%
Low NLL (0–4)	770	31.92%
Moderate NLL (5–7)	866	35.90%
High NLL (8–10)	776	32.17%

WHO-5 < 13 indicates lower well-being. PA categories followed PARS-3 scoring standards. NLL categories were used for descriptive purposes only.

### 3.3 Common method bias and measurement validity

Harman’s single-factor test was conducted as an initial common method bias check. The first unrotated factor explained 17.90% of the variance when the objective nutrition label test and self-report items were analyzed together and 26.38% when only self-report items were included. Both values were below the commonly used 40% threshold, suggesting that common method bias was unlikely to be severe. However, because several variables were assessed by self-report, shared method variance cannot be completely ruled out.

Confirmatory factor analysis was conducted for T1 healthy eating self-efficacy, T2 healthy eating self-efficacy, T1 mental well-being, and T3 mental well-being. The four-factor model showed good fit and was superior to the more constrained alternative models (Table 6). Composite reliability values were acceptable. Although the AVE values were slightly below the conventional 0.50 threshold, HTMT values supported discriminant validity (Tables 7, 8).

**Table 6:** Confirmatory factor analysis model comparison.

Model	χ^2^	df	CFI	TLI	RMSEA	SRMR
Four-factor model	459.66	129	0.973	0.967	0.033	0.024
Three-factor model	1180.68	132	0.913	0.899	0.057	0.044
Two-factor model	1935.05	134	0.851	0.829	0.075	0.059
One-factor model	5487.42	135	0.556	0.497	0.128	0.135

The four-factor model separated T1 HESE, T2 HESE, T1 WHO-5, and T3 WHO-5. The three-factor model combined T1 and T2 HESE; the two-factor model combined all HESE items and all WHO-5 items.

**Table 7:** Composite reliability, AVE, and standardized loadings.

Construct	CR	AVE	Standardized loading range
T1 HESE	0.764	0.448	0.629–0.705
T2 HESE	0.744	0.421	0.615–0.681
T1 WHO5	0.770	0.401	0.606–0.658
T3 WHO5	0.794	0.435	0.634–0.684

CR, composite reliability; AVE, average variance extracted.

**Table 8:** HTMT matrix for discriminant validity.

Construct	T1 HESE	T2 HESE	T1 WHO5	T3 WHO5
T1 HESE	—			
T2 HESE	0.669	—		
T1 WHO5	0.261	0.077	—	
T3 WHO5	0.262	0.332	0.688	—

Lower triangular HTMT values are reported. All values were below 0.85.

Longitudinal measurement invariance of healthy eating self-efficacy across T1 and T2 was then examined. The configural model fit the data well (χ^2^(15) = 33.54, CFI = 0.996, TLI = 0.993, RMSEA = 0.023, SRMR = 0.013). Constraining the factor loadings to equality across waves did not meaningfully worsen model fit (Δχ^2^(3) = 5.12, *p* = 0.163, ΔCFI < 0.001), supporting full metric invariance. Constraining all item intercepts produced a significant decrement in fit (Δχ^2^(3) = 77.37, *p* < 0.001, ΔCFI = 0.015), so full scalar invariance was not supported; after the intercept of one item (the fourth item, which showed the largest change in mean level across the two waves) was freed, the partial scalar model fit the data well (χ^2^(20) = 60.83, CFI = 0.992, TLI = 0.989, RMSEA = 0.029, SRMR = 0.016; ΔCFI = 0.004 relative to the metric model). These results indicate that healthy eating self-efficacy was measured on the same metric across waves, supporting the use of the T1 scores as baseline covariates and the T2 scores as the mediator, although latent mean-level comparisons over time should be interpreted with slight caution.

### 3.4 Correlation analysis

Correlations among covariates and core variables are presented in Table 1. T1 nutrition label literacy was positively correlated with T2 healthy eating self-efficacy and T3 mental well-being. T2 healthy eating self-efficacy was positively correlated with T3 mental well-being. T2 physical activity was positively correlated with both T2 healthy eating self-efficacy and T3 mental well-being, supporting its inclusion as a moderator. The correlation between age and grade was high; therefore, grade was examined only in sensitivity analysis rather than included simultaneously in the main model.

### 3.5 Longitudinal mediation analysis

The longitudinal mediation model adjusted for age, gender, only-child status, residence, T1 healthy eating self-efficacy, and T1 mental well-being. T1 nutrition label literacy was positively associated with T2 healthy eating self-efficacy. T2 healthy eating self-efficacy was positively associated with T3 mental well-being. The direct association between T1 nutrition label literacy and T3 mental well-being remained significant after including T2 healthy eating self-efficacy, indicating partial mediation. The indirect association was significant based on 5,000 bootstrap resamples (Tables 9 and 10). In terms of magnitude, the standardized indirect association (*β* = 0.046) corresponded to approximately 29% of the total association (*β* = 0.157) and should be regarded as small in absolute terms, which is consistent with the conceptual distance between a brief label-comparison test and later psychological well-being. The full model explained 35.1% of the variance in T3 mental well-being, a substantial share of which was attributable to baseline well-being.

**Table 9:** Longitudinal mediation model.

Outcome	Predictor	*β*	SE	*t*	*p*	95% CI	*R* ^2^
T2 HESE	T1 NLL	0.231	0.017	13.49	<0.001	[0.197, 0.265]	0.309
T2 HESE	T1 HESE	0.506	0.017	29.04	<0.001	[0.472, 0.540]	0.309
T3 WHO-5	T1 NLL	0.111	0.017	6.46	<0.001	[0.077, 0.145]	0.351
T3 WHO-5	T2 HESE	0.201	0.020	10.16	<0.001	[0.162, 0.239]	0.351
T3 WHO-5	T1 WHO5	0.523	0.017	30.86	<0.001	[0.490, 0.556]	0.351

Standardized coefficients are shown. Models adjusted for age, gender, only-child status, residence, T1 HESE, and T1 WHO-5 as appropriate. NLL, nutrition label literacy; HESE, healthy eating self-efficacy; WHO-5, WHO-5 Well-Being Index; CI, confidence interval; SE, standard error. *N* = 2,412.

**Table 10:** Total, direct, and indirect effects.

Effect	*β*	SE	95% CI
Total effect	0.157	0.017	[0.124, 0.191]
Direct effect	0.111	0.017	[0.077, 0.145]
Indirect effect	0.046	0.006	[0.036, 0.058]

The indirect effect was estimated with 5,000 bootstrap resamples. Effects are standardized estimates; CI = bootstrap 95% confidence interval. *N* = 2,412.

### 3.6 Moderated mediation analysis

The moderated mediation model further included T2 physical activity and the T2 healthy eating self-efficacy × T2 physical activity interaction term. T2 physical activity was positively associated with T3 mental well-being. The interaction between T2 healthy eating self-efficacy and T2 physical activity was significant, indicating that physical activity moderated the second-stage pathway. The interaction added a small amount of explained variance to the outcome model (ΔR^2^ = 0.015). Although modest in absolute size, this increment indicates that the strength of the indirect pathway varied across levels of physical activity, and its practical relevance lies mainly in identifying for whom the pathway is stronger rather than in producing large mean differences. The index of moderated mediation was significant, supporting a conditional indirect association (Tables 11, 12).

**Table 11:** Moderated mediation regression model.

Outcome	Predictor	*β*	SE	*t*	*p*	95% CI
T3 WHO-5	T1 NLL	0.103	0.017	6.06	<0.001	[0.069, 0.136]
T3 WHO-5	T2 HESE	0.180	0.020	9.15	<0.001	[0.142, 0.219]
T3 WHO-5	T2 PA	0.084	0.017	4.95	<0.001	[0.051, 0.117]
T3 WHO-5	T2 HESE × T2 PA	0.122	0.016	7.51	<0.001	[0.090, 0.153]
T3 WHO-5	T1 WHO5	0.520	0.017	31.24	<0.001	[0.487, 0.552]

Standardized coefficients are shown. The outcome model R^2^ was 0.377; adding the interaction increased *R*^2^ by 0.015. NLL, nutrition label literacy; HESE, healthy eating self-efficacy; PA, physical activity; WHO-5, WHO-5 Well-Being Index; CI, confidence interval; SE = standard error. *N* = 2,412. Covariates: age, gender, only-child status, residence, T1 HESE, and T1 WHO-5.

**Table 12:** Index of moderated mediation and conditional indirect effects.

Effect/condition	*β*	SE	95% CI
Index of moderated mediation	0.028	0.004	[0.020, 0.037]
Low PA (−1 SD)	0.014	0.006	[0.002, 0.025]
Mean PA	0.042	0.005	[0.032, 0.053]
High PA (+1 SD)	0.070	0.008	[0.056, 0.086]

PA values represent standardized physical activity levels. Bootstrap 95% confidence intervals were based on 5,000 resamples. *N* = 2,412; SE, standard error.

### 3.7 Simple slope and conditional indirect effect analyses

**Figure 2:**
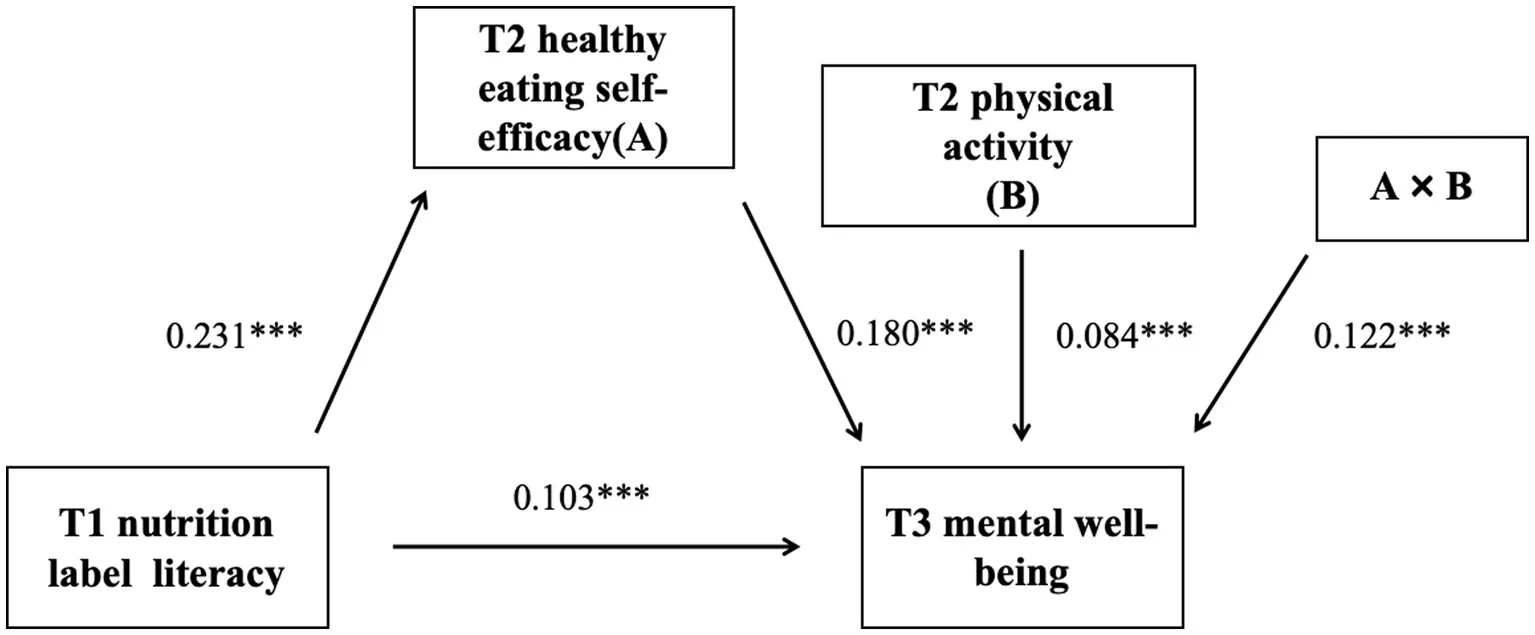
Statistical longitudinal moderated mediation model with standardized coefficients.

**Figure 3:**
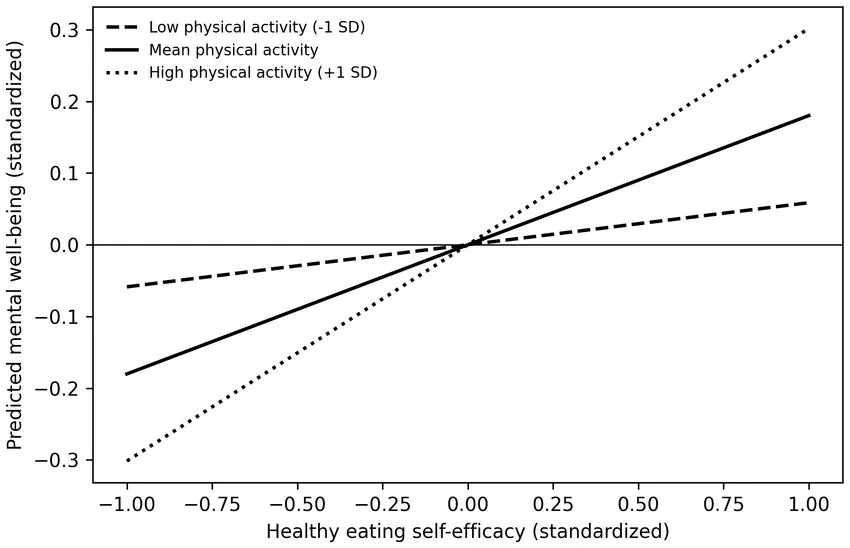
Simple slopes of the association between *T2* healthy eating self-efficacy and *T3* mental well-being across levels of *T2* physical activity.

**Figure 4:**
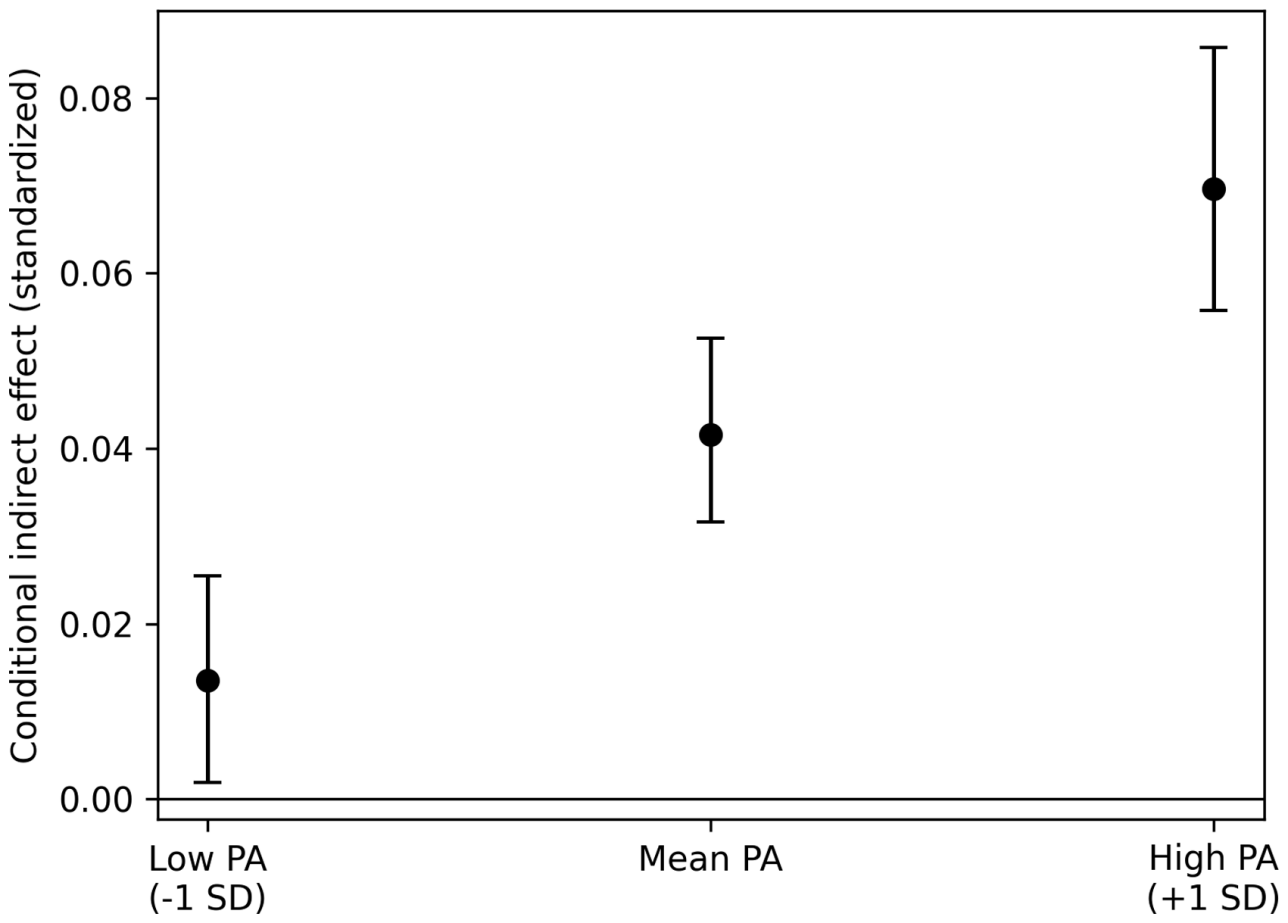
Conditional indirect effects of T1 nutrition label literacy on T3 mental well-being through T2 healthy eating self-efficacy across levels of T2 physical activity.

Simple slope analysis showed that T2 healthy eating self-efficacy was positively associated with T3 mental well-being at low, mean, and high levels of T2 physical activity, but the association was stronger at higher physical activity levels. Conditional indirect effects also increased across physical activity levels, with the strongest indirect association observed at high physical activity (Table 13; Figures 2–4).

**Table 13:** Simple slopes for the association between T2 HESE and T3 WHO-5.

Physical activity level	β	SE	t	p	95% CI
Low PA (−1 SD)	0.059	0.025	2.32	0.021	[0.009, 0.108]
Mean PA	0.180	0.020	9.15	<0.001	[0.142, 0.219]
High PA (+1 SD)	0.302	0.026	11.75	<0.001	[0.251, 0.352]

Simple slopes were calculated from the moderated mediation model at standardized values of physical activity. *N* = 2,412; CI, confidence interval.

### 3.8 Sensitivity analyses

Sensitivity analyses were conducted by excluding participants who failed the attention-check item, excluding participants who completed any wave in less than 120 s, excluding both types of data-quality risk, and additionally controlling for grade. The substantive pattern of results remained unchanged across all sensitivity analyses (Table 14). Because age and grade were highly correlated, grade was not included in the main model but was included as an additional sensitivity covariate. VIF values in the main model were all below 1.50, indicating no meaningful multicollinearity. When age and grade were entered simultaneously, VIF values for age and grade exceeded 6, confirming that they should not be included together in the primary model.

**Table 14:** Sensitivity analyses.

Analysis	*n*	T1 NLL → T2 HESE	T2 HESE → T3 WHO-5	Interaction	Index
Main model	2,412	0.231	0.180	0.122	0.028
Excluding attention-check failures	2,320	0.232	0.177	0.121	0.028
Excluding response time <120 s	1956	0.228	0.180	0.136	0.031
Excluding both data-quality risks	1882	0.232	0.178	0.134	0.031
Additionally controlling grade	2,412	0.231	0.180	0.121	0.028

Values are standardized coefficients or standardized index estimates. All listed focal parameters remained statistically significant in the same direction as the main model. NLL, nutrition label literacy; HESE, healthy eating self-efficacy; WHO-5, WHO-5 Well-Being Index; Index, index of moderated mediation.

## 4 Discussion

This three-wave longitudinal study examined whether nutrition label literacy was prospectively associated with mental well-being among Chinese adolescents through healthy eating self-efficacy and whether the association between healthy eating self-efficacy and later well-being differed across levels of physical activity. The findings supported the proposed moderated mediation model. T1 nutrition label literacy was positively associated with T2 healthy eating self-efficacy, which in turn was positively associated with T3 mental well-being after baseline well-being was controlled. Higher physical activity was associated with a stronger association between healthy eating self-efficacy and later well-being, and the indirect association was stronger at higher levels of physical activity. These findings should be interpreted as longitudinal associations rather than causal effects.

### 4.1 Nutrition label literacy and mental well-being

The positive longitudinal association between nutrition label literacy and later mental well-being extends nutrition literacy research beyond knowledge, label use, and dietary outcomes. Previous work has defined food literacy and nutrition literacy as practical capacities that allow individuals to access, interpret, evaluate, and apply food-related information in real-life decision-making contexts. From this perspective, adolescents who are better able to compare nutrition labels may not only identify healthier packaged foods but also experience a stronger sense of competence when navigating complex food environments. This interpretation is consistent with research showing that nutrition knowledge and label use are related to dietary choices, although these links are usually modest and context dependent (66). Evidence from adolescent food label literacy research further suggests that label reading and label comprehension are not interchangeable; adolescents may report reading labels while still having limited ability to evaluate nutrition information accurately. The present findings therefore support a label-specific literacy perspective rather than a broad claim that general nutrition knowledge is sufficient for healthier functioning.

The finding also fits the developmental context of adolescence. Food choices during this period are shaped by peer norms, convenience, school routines, family meals, food availability, and marketing exposure. Neighborhood food-environment research also indicates that access to healthier foods differs across settings, which can shape how literacy is used (67). School-based consumption studies show that low-nutrient, energy-dense foods are obtained in multiple locations, not only outside school (68). Nutrition label literacy may help adolescents reduce uncertainty when judging packaged foods, especially in environments where food claims, processing levels, and nutrient information are not always easy to interpret (69). Young adult research has also linked health literacy, food label use, and diet-related self-management, suggesting that label-related competence can be connected with broader health behavior capacities (70). This capability may support perceived control and health-oriented decision-making, both of which are relevant to positive psychological functioning. However, the direct path from T1 nutrition label literacy to T3 mental well-being remained small, indicating that the association is likely distal and partly dependent on intervening psychosocial processes. This pattern is consistent with adolescent health literacy research showing that literacy is related to health behavior and psychosocial outcomes, but rarely operates as a single dominant determinant. Childhood and youth health literacy models also emphasize that literacy is embedded in school, family, community, and media environments, which may shape how literacy is converted into everyday choices.

The result further broadens the literature connecting diet-related constructs with adolescent mental well-being. Prior longitudinal studies have suggested that diet quality and specific dietary behaviors may be associated with later mental health outcomes in youth. Early-adolescent dietary-pattern research has linked diet quality with later mental health indicators (71). Systematic reviews also indicate that diet–mental health associations in children and adolescents are plausible but heterogeneous across study designs, measurements, and populations. Reviews focused on diet and depression in youth have similarly emphasized that evidence is suggestive but not deterministic. Broader psychological well-being research has long treated positive affect, vitality, and daily engagement as meaningful indicators of healthy functioning rather than secondary outcomes. Health psychology likewise emphasizes that well-being can be shaped by perceived control, social conditions, and lifestyle-related resources. In the current study, nutrition label literacy was not a direct measure of dietary intake, which means that its association with well-being should be interpreted through the lens of food-related competence and decision-making capacity rather than nutrient consumption itself. In this sense, nutrition label literacy may represent a cognitive resource associated with healthier decision-making, but the behavioral mechanisms underlying this association require further investigation because actual dietary intake was not assessed in this study. Future studies should test whether actual food label use, dietary quality, and ultra-processed food consumption further explain the link between label literacy and well-being.

### 4.2 Mediating role of healthy eating self-efficacy

Healthy eating self-efficacy partially mediated the longitudinal association between nutrition label literacy and mental well-being. This result supports social cognitive theory, which emphasizes that knowledge and skills become behaviorally meaningful when individuals believe they can perform relevant actions in everyday contexts. The broader self-efficacy framework also suggests that perceived capability is closely tied to motivation, persistence, and emotional functioning (72). Adolescents may understand nutrition labels but still struggle to choose healthier foods when faced with taste preferences, time pressure, school food options, peer eating norms, or convenience products. Health behavior theory therefore treats self-efficacy as a proximal determinant that can translate information and skills into more adaptive behavior (73). The significant T1 nutrition label literacy to T2 healthy eating self-efficacy path suggests that label-related competence may be associated with stronger confidence in making healthier choices over time.

The mediator also helps explain why the relation between label literacy and well-being should not be treated as a simple direct association. Previous adolescent research has shown that healthy eating self-efficacy is associated with perceived control, food choice confidence, peer context, and health-related attitudes. Additional work on children’s eating self-regulation supports the idea that eating-related self-efficacy is relevant when individuals must manage temptation, low energy, negative emotion, or competing environmental cues (74). Focus-group research with adolescents has shown that convenience, taste, social situations, and time pressure frequently shape food choices more immediately than abstract nutrition knowledge. This means that self-efficacy is important because it reflects whether adolescents believe they can use nutrition knowledge under real-world constraints. In the present study, T2 healthy eating self-efficacy was associated with T3 mental well-being after baseline well-being and baseline self-efficacy were controlled. This finding suggests that self-efficacy may function as a proximal psychosocial bridge connecting an upstream literacy resource with a later positive health outcome.

The observed mediation should nevertheless be interpreted cautiously. The indirect effect was statistically significant, but its magnitude was modest, which is appropriate given the conceptual distance between a label-literacy task and later psychological well-being. Diet–mental health research has repeatedly emphasized that the associations between dietary factors and adolescent psychological outcomes are usually shaped by multiple biological, psychological, family, school, and socioeconomic influences. Dietary patterns in adolescence can also show developmental stability, meaning that short-term changes in confidence may operate against relatively established food routines (75). Prior prospective evidence linking diet quality with adolescent mental health supports the temporal plausibility of the model, but it does not imply that a label-literacy task alone can determine later well-being. Healthy eating self-efficacy may therefore be one pathway rather than the only pathway. Other mediators, such as actual label use, breakfast regularity, fruit and vegetable intake, ultra-processed food consumption, perceived dietary control, or family food support, may also contribute to the association (76). Future longitudinal studies could build a more detailed chain model linking nutrition label literacy to self-efficacy, actual dietary behavior, and well-being.

### 4.3 Moderating role of physical activity

Higher physical activity was associated with a stronger positive association between healthy eating self-efficacy and subsequent mental well-being. This pattern is consistent with youth physical activity evidence showing that movement behaviors are related to psychological functioning, affective regulation, cognitive resources, and perceived competence. Reviews of physical activity and mental health in children and adolescents have also suggested that active lifestyles are associated with more favorable psychological outcomes, although the magnitude of associations is often modest. In the current model, physical activity did not replace healthy eating self-efficacy as a mechanism; rather, it provided a behavioral context in which confidence in healthy eating was more strongly associated with later well-being. Adolescents who are more physically active may experience greater vitality, better stress recovery, and a stronger sense of mastery, which may make the psychological benefits of healthy eating confidence more salient.

The moderation result also supports a multidomain lifestyle interpretation. Related evidence from broader physical-activity, physical-education, and health-promotion research also supports this contextual interpretation: physical activity has been linked with depressive symptoms, physical education models emphasize school-based activity routines, and community health-promotion work connects health-supportive practices with sustainable well-being (77–79). Nutrition-related self-efficacy may be more likely to translate into well-being when embedded in broader patterns of health-oriented behavior. School-based healthy eating intervention evidence suggests that nutrition education is more promising when it addresses multiple determinants rather than knowledge alone. Physical activity guidelines similarly emphasize that movement behavior is a core component of adolescent health promotion. Objective physical activity research has linked activity to multiple health indicators in school-aged youth, supporting the idea that movement behavior reflects a broad health-development context. Evidence on physical activity benefits in school-aged youth further indicates that active behavior is associated with physical, metabolic, cognitive, and psychological outcomes. In this context, high physical activity may indicate a wider pattern of self-regulation, routine formation, and engagement in health-promoting activities. This may explain why the conditional indirect effect was stronger at higher levels of physical activity.

At the same time, the moderation finding should not be overstated. The study was observational, and the interaction term should be interpreted as a conditional longitudinal association rather than proof that physical activity amplifies the effect of healthy eating self-efficacy. Recent longitudinal evidence suggests that the total and temporal patterning of physical activity may be associated with later adolescent well-being, but these associations remain probabilistic rather than deterministic. The additional explanatory contribution of interaction effects is often small in longitudinal behavioral research, and the present finding should be described as modest but meaningful. It is also possible that physical activity is a marker of other unmeasured resources, such as supportive school climate, parental encouragement, peer group norms, sleep regularity, or lower academic stress. Future intervention studies are needed to determine whether programs that combine food-label education, self-efficacy enhancement, and physical activity promotion produce stronger well-being benefits than nutrition education alone.

### 4.4 Theoretical and practical implications

Theoretically, this study connects nutrition label literacy with adolescent mental well-being through a longitudinal psychosocial pathway. It extends food label literacy research from dietary decision-making alone to positive psychological functioning. The findings also support the relevance of social cognitive theory in adolescent nutrition research by showing that healthy eating self-efficacy may help explain how label-related competence is linked to later well-being. In addition, the moderating role of physical activity supports a multidomain lifestyle perspective, in which nutrition education and movement promotion are treated as complementary components of school health promotion.

Practically, the findings suggest that school nutrition education should go beyond explaining nutrients in isolation. Programs may be more useful when they teach adolescents how to compare labels, identify ingredients, interpret sugar, sodium, fiber, and whole-grain information, and apply these skills in realistic food-choice situations. Nutrition education may also benefit from self-efficacy-building strategies, such as guided practice, goal setting, peer discussion, and scenario-based decision-making. The physical activity findings suggest that nutrition education may be most meaningful when integrated with broader school health-promotion efforts, including physical education, active breaks, and supportive family and school environments. However, because the study was observational, these implications should be treated as directions for future intervention research rather than proof of intervention effectiveness.

### 4.5 Limitations and future research

Several limitations should be noted. The study used a three-wave longitudinal design, but it was observational and cannot establish strict causality. In addition, the intervals between waves were approximately 2 months each, so the total follow-up period of about 4 months may be too short to capture longer-term psychological change, and the observed associations may partly reflect relatively short-term covariation. All variables were assessed by self-report or task-based questionnaire methods, which may introduce response bias. Although Harman’s single-factor test did not suggest severe common method bias, most variables relied on questionnaire data collected from the same respondents, so common method variance cannot be fully ruled out and may have inflated some of the observed associations. Observed-score regression analyses also do not fully correct for measurement error. The nutrition label literacy test was adapted and contextualized for Chinese packaged-food labels; however, no formal expert panel review, pilot testing, cognitive interviews, or standardized translation–back-translation procedures were conducted during the adaptation. Although the test showed acceptable reliability, appropriate item difficulty, adequate item discrimination, and theoretically expected correlations in this sample, further validation with expert content review, cognitive interviewing, and item response theory methods is warranted. The study did not include objective dietary intake, actual food label use, or ultra-processed food consumption, which limits conclusions about behavioral pathways. Physical activity was measured with a brief behavioral index rather than objective accelerometry. The sample was school-based and may not represent all Chinese adolescents. In particular, participants were recruited through convenience sampling from 10 schools with established research collaborations in central, northern, and southern China, and rural students accounted for approximately 70% of the sample, so selection bias is possible and the findings may not generalize to adolescents in other regions or school types. Several potentially important confounders were also not measured, including family socioeconomic status, parental education, the family dietary environment, and academic stress, so residual confounding cannot be ruled out. Moreover, full scalar invariance of the healthy eating self-efficacy measure was not supported; although partial scalar invariance was achieved, mean-level comparisons over time should be interpreted with slight caution. Future studies should include more diverse regions, longer follow-up periods, objective movement measures, actual dietary indicators, and intervention designs that test whether improving nutrition label literacy and physical activity can change self-efficacy and well-being.

## 5 Conclusion

This three-wave longitudinal study found that nutrition label literacy was prospectively associated with later mental well-being among Chinese adolescents, partly through healthy eating self-efficacy. Higher physical activity was associated with a stronger positive association between healthy eating self-efficacy and later mental well-being, and the indirect association was stronger at higher levels of physical activity. These findings suggest that integrated school-based strategies combining nutrition label education, healthy eating self-efficacy enhancement, and physical activity promotion are promising, but their effectiveness should be evaluated in future intervention studies. The conclusions should be interpreted as longitudinal associations rather than causal effects.

## Data Availability

The datasets generated and/or analyzed during the current study are not publicly available due to ethical and confidentiality considerations related to the participation of minors. De-identified data may be made available by the corresponding author upon reasonable request, subject to applicable ethical and confidentiality requirements.

## Appendix

**Table A1:** Item-level difficulty and corrected item-total discrimination for the 10-item nutrition label literacy test (final matched sample, *N* = 2,412).

Item	Difficulty (p)	Corrected item-total discrimination
1	0.469	0.401
2	0.682	0.401
3	0.575	0.420
4	0.459	0.401
5	0.576	0.428
6	0.621	0.397
7	0.467	0.420
8	0.617	0.400
9	0.536	0.411
10	0.804	0.398

Difficulty (p) is the proportion of correct responses. Discrimination is the corrected item-total (point-biserial) correlation. Internal consistency (KR-20) was 0.747.
